# Ecos do Passado: A Intrigante Ligação entre a Doença de Chagas e a Insulina

**DOI:** 10.36660/abc.20250295

**Published:** 2025-06-02

**Authors:** Marcia M. Noya-Rabelo, Olga Souza

**Affiliations:** 1 Instituto D’Or de Ensino e Pesquisa Salvador BA Brasil Instituto D’Or de Ensino e Pesquisa (IDOR), Salvador, BA – Brasil; 2 Escola Bahiana de Medicina e Saúde Pública Salvador BA Brasil Escola Bahiana de Medicina e Saúde Pública – EBSMP, Salvador, BA – Brasil; 3 Hospital Aliança Salvador BA Brasil Hospital Aliança, Salvador, BA – Brasil

**Keywords:** Insulina, Doença de Chagas

A doença de Chagas é atualmente classificada como uma das várias doenças tropicais negligenciadas e como uma das cinco infecções parasitárias negligenciadas priorizadas pela Organização Mundial da Saúde e pelo Centro de Controle de Doenças para intervenção em saúde pública.^1^ Isso ressalta o fato de que o agente causador da tripanossomíase americana existe há aproximadamente 9.000 anos, com amostras positivas datando de 7050 a.C.^2^ O ciclo de vida do Trypanosoma cruzi (T. cruzi) compreende diversas transformações morfológicas envolvendo hospedeiros mamíferos e vetores, onde três diferentes estágios principais de desenvolvimento são identificados: epimastigotas, tripomastigotas e amastigotas.^3^ Tradicionalmente, sabe-se que o T. cruzi tem como alvo o coração e o trato gastrointestinal; no entanto, seu parasitismo também resulta em alterações estruturais e efeitos pró-inflamatórios no tecido adiposo e no pâncreas.

Nesta edição dos Arquivos Brasileiros de Cardiologia, um estudo longitudinal de Barbosa-Ferreira et al.^4^ avaliaram os níveis de insulina, adiponectina e leptina antes e depois do tratamento da doença de Chagas aguda com benznidazol. Vinte e oito indivíduos foram divididos em dois grupos: um grupo controle (15 indivíduos) e um grupo com doença de Chagas aguda (13 indivíduos). Os autores não encontraram diferenças significativas nos níveis séricos de adiponectina e leptina entre os grupos. Em contraste, os níveis séricos de insulina foram menores no grupo com doença de Chagas aguda, tanto antes quanto depois do tratamento, em comparação ao grupo controle. Além disso, os níveis de insulina foram menores na fase pós-tratamento em comparação à fase pré-tratamento.

Vários estudos examinaram as consequências da infecção por T. cruzi na função pancreática em roedores. Lenzi et al.^5^ usou camundongos BALB/c infectados com a cepa CL de T. cruzi e sacrificados 15 dias após a infecção. Havia pancreatite intersticial difusa associada a edema, infiltração de monócitos e macrófagos, especialmente ao redor dos ductos intralobulares, e atrofia lobular, sugerindo perda de células acinares. Por outro lado, outro grupo encontrou ninhos de amastigotas em ilhotas, bem como em ductos pancreáticos e ácinos.^6^ A presença de amastigotas no pâncreas endócrino e exócrino foi confirmada em camundongos infectados em um estudo separado.^7^

O grupo de Santos et al.^8^ empregando um modelo de hamster, investigou as consequências da infecção por T. cruzi nas células β pancreáticas. Os níveis de glicose sanguínea e insulina circulantes foram coletados dos animais durante a fase aguda, bem como na fase crônica. As concentrações plasmáticas de insulina foram significativamente menores em 6 dos 8 animais infectados, tanto na fase aguda quanto na crônica, em comparação com os animais controle não infectados, de modo que, em geral, os animais infectados apresentaram níveis de insulina mais baixos em comparação aos controles. A hipoinsulinemia já havia sido descrita por outros autores em modelos murinos, e as causas podem ser multifatoriais, como consequência do parasitismo do pâncreas e da inflamação, fibrose e atrofia do parênquima subsequente; insulinite;^8^ ganglionite intrapancreática;^9^ anormalidades na inervação pancreática, especialmente envolvendo o sistema nervoso parassimpático;^8 , 10^ e alteração do eixo enteroinsular relacionada à disfunção autonômica.^10^

O papel do T. cruzi na regulação da secreção de insulina durante a infecção de células β não foi estudado extensivamente em humanos ou em modelos animais. A revisão dos estudos atuais em humanos sobre se a infecção por T. cruzi leva à hiperglicemia e diabetes, por outro lado, é inconclusiva. Além disso, a diminuição da secreção de insulina não se correlacionou consistentemente com o aumento da prevalência de hiperglicemia.^11 , 12^

Até o momento, nossa compreensão da secreção hormonal em pacientes com doença de Chagas derivou principalmente de um número limitado de estudos clínicos, muitos dos quais com amostras pequenas. Este artigo oferece novos insights sobre a complexa fisiopatologia da doença de Chagas, adicionando informações sobre os níveis de insulina durante a fase aguda em humanos.^4^

Também precisamos examinar em que medida a regulação e as ações do Trypanosoma cruzi são redundantes ou únicas em diferentes órgãos-alvo. É crucial avaliar se mecanismos de controle seletivo em diferentes tecidos estão envolvidos, exigindo coordenação em um nível mais global. Por exemplo, considerar o papel da abundância e da sensibilidade à insulina como mediadores de interações hospedeiro-parasita de amplo alcance pode abrir caminho para novas estratégias de controle parasitário.
