# Supplementary material for: Ecos do Passado: A Intrigante Ligação entre a Doença de Chagas e a Insulina
Source: Arq Bras Cardiol. 2025 Jun 2;(5):e20250295. [Article in Portuguese] doi: 10.36660/abc.20250295 (PMC12212383; doi:10.36660/abc.20250295)
Supplement: Translation

# Translation: Echoes of the Past: The Intriguing Link between Chagas Disease and Insulin

**DOI:** 10.36660/abc.20250295i

**Authors:** Marcia M. Noya-Rabelo, Olga Souza

**Affiliations:** 1 Salvador BA Brazil Instituto D’Or de Ensino e Pesquisa (IDOR), Salvador, BA – Brazil; 2 Salvador BA Brazil Escola Bahiana de Medicina e Saúde Pública – EBSMP, Salvador, BA – Brazil; 3 Salvador BA Brazil Hospital Aliança, Salvador, BA – Brazil

**Keywords:** Insulin, Acute Chagas Disease

Chagas disease is currently classified as one of several neglected tropical diseases and as one of the five neglected parasitic infections prioritized by the World Health Organization and the Center for Disease Control for public health intervention.^1^This underscores the fact that the causative agent of American trypanosomiasis has existed for approximately 9.000 years, with positive samples dating back to 7050 B.C.^2^The life cycle of *Trypanosoma cruzi (T. cruzi)* comprises several morphological transformations involving both mammalian and vector hosts, where three different major developmental stages are identified: epimastigotes, trypomastigotes, and amastigotes.^3^Traditionally, *T. cruzi* is known to target the heart and gastrointestinal tract; however, its parasitism also results in structural changes and pro-inflammatory effects in adipose tissue and the pancreas.

In this edition of the *Arquivos Brasileiros de Cardiologia* , a longitudinal study by Barbosa-Ferreira et al.^4^evaluated the levels of insulin, adiponectin, and leptin before and after treatment of acute Chagas disease with benznidazole. Twenty-eight subjects were divided into two groups: a control group (15 subjects) and an acute Chagas disease group (13 subjects). The authors found no significant differences in serum levels of adiponectin and leptin between the groups. In contrast, serum levels of insulin were lower in the acute Chagas group, both before and after treatment, compared to the control group. Additionally, insulin levels were lower in the post-treatment phase compared to the pre-treatment phase.

Several studies have examined the consequences of *T. cruzi* infection on pancreatic function in rodents. Lenzi et al.^5^infected BALB/c mice with the CL strain of *T. cruzi* and sacrificed 15 days post-infection. There was diffuse interstitial pancreatitis associated with edema, infiltration of monocytes and macrophages, especially around intralobular ducts, and lobular atrophy, suggesting acinar cell loss. On the other hand, another group found amastigote nests in islets as well as pancreatic ducts and acini.^6^Presence of amastigotes in both the endocrine and exocrine pancreas was confirmed in infected mice in a separate study.^7^

The Santos et al.^8^group, employing a hamster model, investigated the consequence of *T. cruzi* infection on pancreatic β cells. Blood glucose and insulin levels in circulation were sampled in the animals during the acute phase as well as in the chronic phase. Plasma insulin concentrations were significantly lower in 6 out of 8 infected animals during both acute and chronic infection compared to uninfected control animals, so that overall, infected animals displayed lower insulin levels compared to controls. Hypoinsulinemia had already been described by other authors in murine models, and the causes may be multifactorial, such as a consequence of parasitism of the pancreas and of inflammation, fibrosis and atrophy of the subsequent parenchyma; insulinitis;^8^intrapancreatic ganglionitis;^9^abnormalities in pancreatic innervation, especially involving the parasympathetic nervous system;^8 , 10^and alteration of the enteroinsular axis related to autonomic dysfunction.^10^

The role of *T. cruzi* in regulating insulin secretion during infection of β cells has not been studied extensively in either humans or animal models. Review of the current human studies as to whether *T. cruzi* infection leads to hyperglycemia and diabetes, on the other hand, is inconclusive. In addition, decreased insulin secretion did not consistently correlate with increased prevalence of hyperglycemia.^11 , 12^

To date, our understanding of hormone secretion in patients with Chagas disease has primarily been derived from a limited number of clinical studies, many of which have small sample sizes. This article offers new insights into the complex pathophysiology of Chagas disease by adding information on insulin levels during the acute phase in humans.^4^

We also need to examine the extent to which the regulation and actions of *Trypanosoma cruzi* are either redundant or unique across different target organs. It is crucial to address whether selective control mechanisms in various tissues are involved, requiring coordination on a more global level. For instance, considering the role of insulin abundance and sensitivity as mediators of broad-range host-parasite interactions could pave the way for new strategies in parasitic control.
